# Ultrasound-based prediction model for years to peak height velocity using multiple secondary ossification centers

**DOI:** 10.1007/s10396-025-01571-y

**Published:** 2025-10-24

**Authors:** Kosuke Uemura, Mizue Saita, Koji Wagatsuma, Wataru Iwamoto, Daichi Morikawa, Yoshimasa Saigo, Toshio Naito

**Affiliations:** 1 Department of General Medicine, Faculty of Medicine, Juntendo University, 3-1-3 Hongo, Bunkyo-Ku, Tokyo 113-8421 Japan; 2 Rehabilitation Unit, Jinseikai, Tokyo, Japan; 3 Department of Sports Medicine and Orthopedics, Edogawa Hospital, Tokyo, Japan; 4 Department of Orthopedics, Faculty of Medicine, Juntendo University, Tokyo, Japan

**Keywords:** Ultrasound, Peak height velocity age, Ossification

## Abstract

**Purpose:**

Determining peak height velocity age (PHVA) is crucial for understanding child growth and development and preventing injuries. Previous studies have used anthropometric measurements or X-ray evaluation to predict the timing of PHV, whereas ultrasound provides a radiation-free and portable alternative. This study aimed to predict the years to PHV by assessing multiple secondary ossification centers using ultrasound.

**Methods:**

A total of 12 sites across eight bones were evaluated using ultrasound in 181 children aged 6–12 years between June and December 2019. Height data were tracked from school entry until December 2022, with PHVA calculated using AUXAL software. Multivariable regression analysis was performed using bone maturity as the explanatory variable and the difference between ultrasound measurement age and PHVA as the dependent variable.

**Results:**

A total of 159 participants were included in the final analysis. The hook of the hamate, calcaneus plantar thickness, plantar sesamoid, and tibial tuberosity were identified as significant variables for PHV prediction. The prediction equation was: Years to PHV = 1.206 + (0.562 × calcaneus plantar thickness) – (1.120 × plantar sesamoid) – (0.675 × tibial tuberosity) + (0.229 × hook of the hamate). This model achieved an adjusted *R*^2^ of 0.782.

**Conclusion:**

Ultrasound evaluation of multiple secondary ossification centers may provide a valuable method for predicting years to PHV.

**Supplementary Information:**

The online version contains supplementary material available at 10.1007/s10396-025-01571-y.

## Introduction

Differences in birth month and individual peak height velocity age (PHVA) have been associated with varying injury risks across multiple studies [1–5]. Therefore, accurately understanding individual growth is crucial from the perspectives of child development and injury prevention. Anthropometric methods, calculation methods, and X-ray bone evaluation are commonly used to predict skeletal age and PHVA, with X-ray evaluation considered the most reliable [6, 7]. Unlike X-ray imaging, ultrasound provides a radiation-free and portable alternative for assessing skeletal maturity in children.

Previous research has suggested that ultrasound evaluation of ossification centers may assist in predicting height growth [8], but that study was limited to semi-quantitative assessment of a single ossification center. The aim of the present study was to predict years to PHV by evaluating multiple secondary ossification centers using ultrasound.

## Materials and methods

### Study design, setting, and participants

**Fig. 1:**
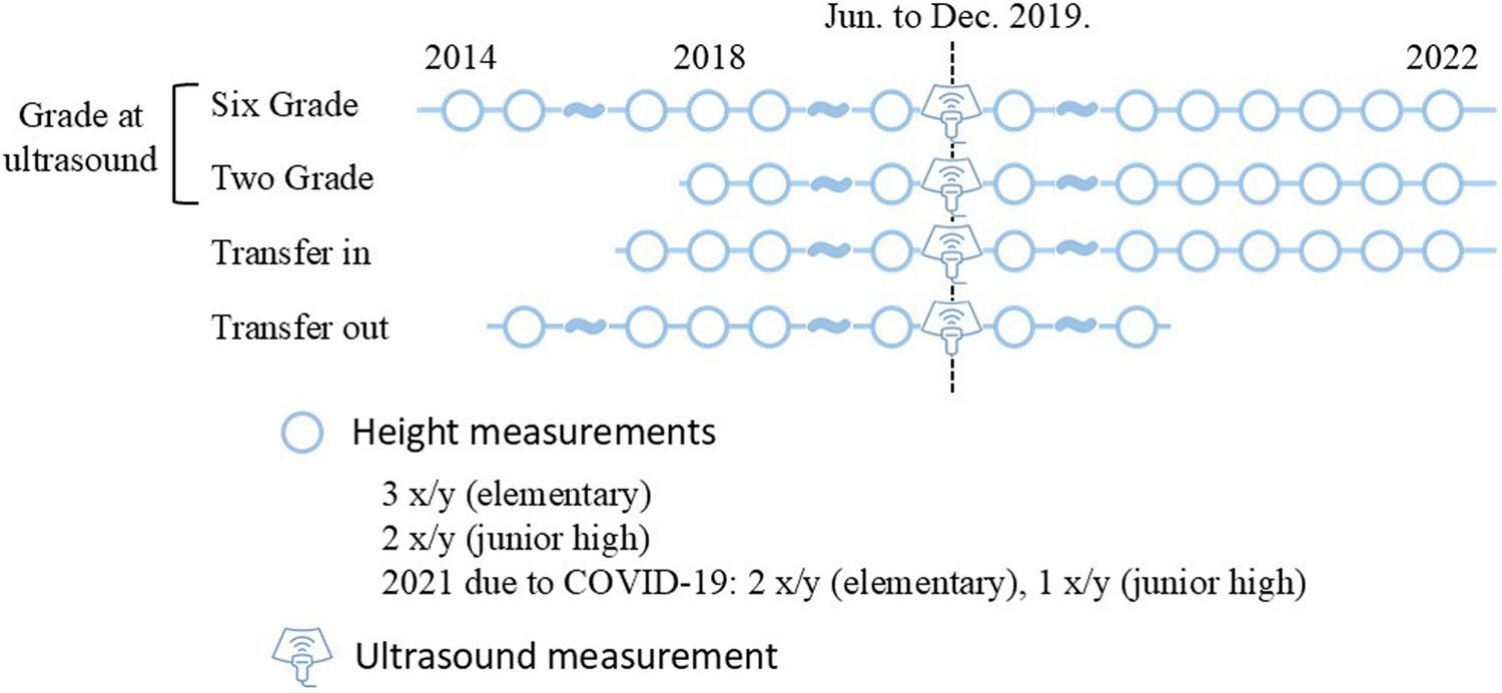
Timeline of height tracking and ultrasound measurements

This cohort study included 181 consenting children aged 6–12 years from a combined elementary and junior high school in Japan. Ultrasound measurements of multiple secondary ossification centers were performed once between June and December 2019. Height data were tracked from elementary school entry until December 2022 (Fig. 1).

### Data source

Height measurements were taken three times annually during regular school check-ups in elementary school. After entering junior high school, measurements were taken twice a year, once during the regular check-up and once by the researchers approximately 6 months later. Height was measured to the nearest 0.1 cm.

The follow-up duration varied depending on the participant’s school grade at the time of the ultrasound examination in 2019. For example, children who were in first grade in 2019 had height data recorded until the fourth grade (approximately 3–4 years), whereas children who were in the sixth grade in 2019 had data spanning from first grade to ninth grade (approximately 8 years).

Ultrasound measurements were performed by a single examiner. The measurement sites included 12 locations across eight bones in the left upper and lower limbs: thumb sesamoid, pisiform, medial epicondyle, plantar sesamoid, tibial tuberosity, hook of the hamate, olecranon (two sites), and calcaneus (four sites). These sites were chosen because they can be easily measured while clothed and are reported to develop ossification centers from several years to several months before the growth period [9]. The left side was chosen to align with X-ray bone age assessment methods [10]. Detailed ultrasound imaging methods are shown in Table 1. To assist in the reproducibility of ultrasound measurements, Supplementary Fig. 1 provides images demonstrating probe orientation and placement relative to anatomical landmarks.

**Table 1:**
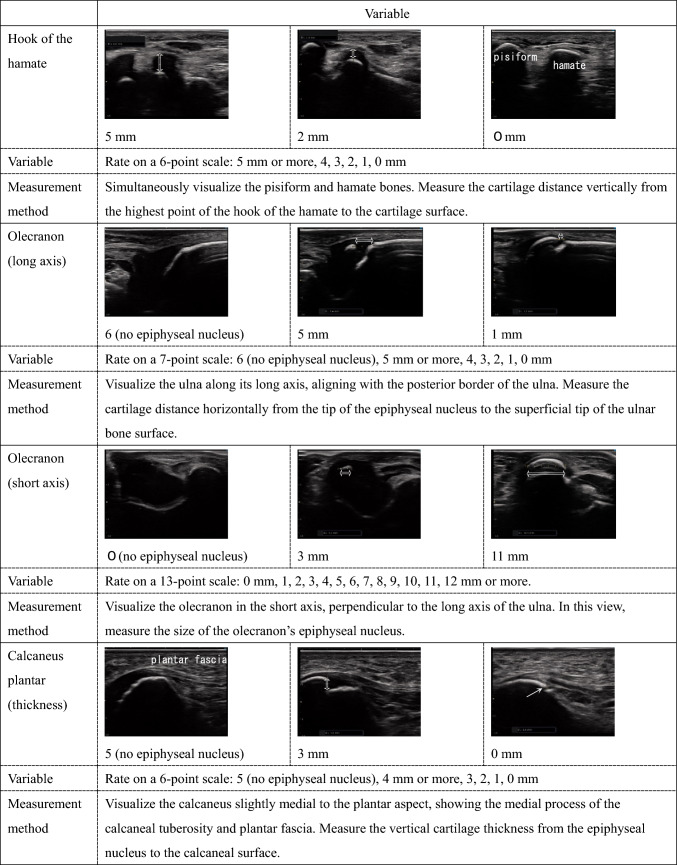

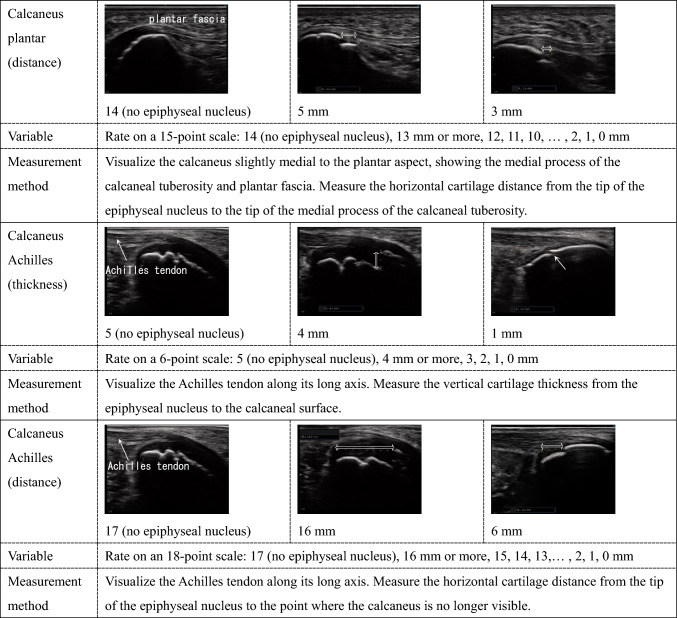
Ordinal variables

For the seven sites that could be quantitatively evaluated (hook of the hamate, olecranon (two sites), and calcaneus (four sites)), the size of the ossification center, cartilage thickness, and distance to epiphyseal closure were measured in millimeters and rounded to the nearest integer for ordinal variable evaluation. The upper limit of measurement values was determined by the researcher when the ossification center size was too large or the distance to epiphyseal closure was too long to ensure reproducibility.

Table 2 shows binary variable evaluations for five ossification centers (thumb sesamoid, pisiform, medial epicondyle, plantar sesamoid, tibial tuberosity) where quantitative assessment was difficult or deemed not meaningful for predicting the timing of PHV.

**Table 2:**
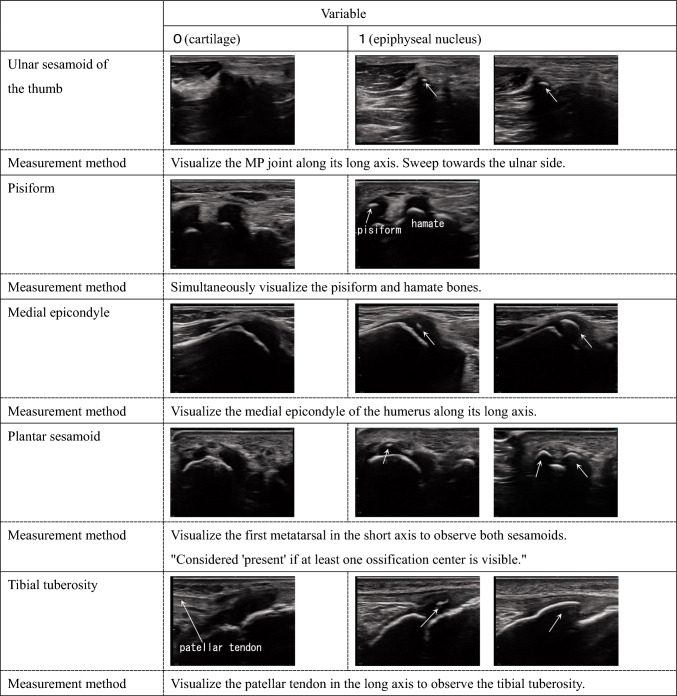
Binary variables

MP: metacarpophalangeal（joint of the thumb)

### Sample size

Although this study was exploratory in nature, the aim was to collect at least 150 samples to account for a maximum of 12 explanatory variables across eight bones and 12 sites [11].

### Statistical analysis

Individual height data were input into the AUXAL software (Scientific Software International, Skokie, IL, USA) to calculate each child’s PHVA. The AUXAL 3.1 program, which can estimate PHVA even if the child has not yet reached it, uses BTT and JPA2 models to fit growth curves and generate velocity curves [12–14]. The difference between the calculated PHVA and the age at ultrasound measurement (years to PHV) was calculated. Multivariable regression analysis was performed using IBM SPSS Statistics version 27.0, with ultrasound-assessed bone maturity (ordinal and binary variables) as explanatory variables and years to PHV (PHVA—Age at ultrasound) as the dependent variable.

## Results

**Fig. 2:**
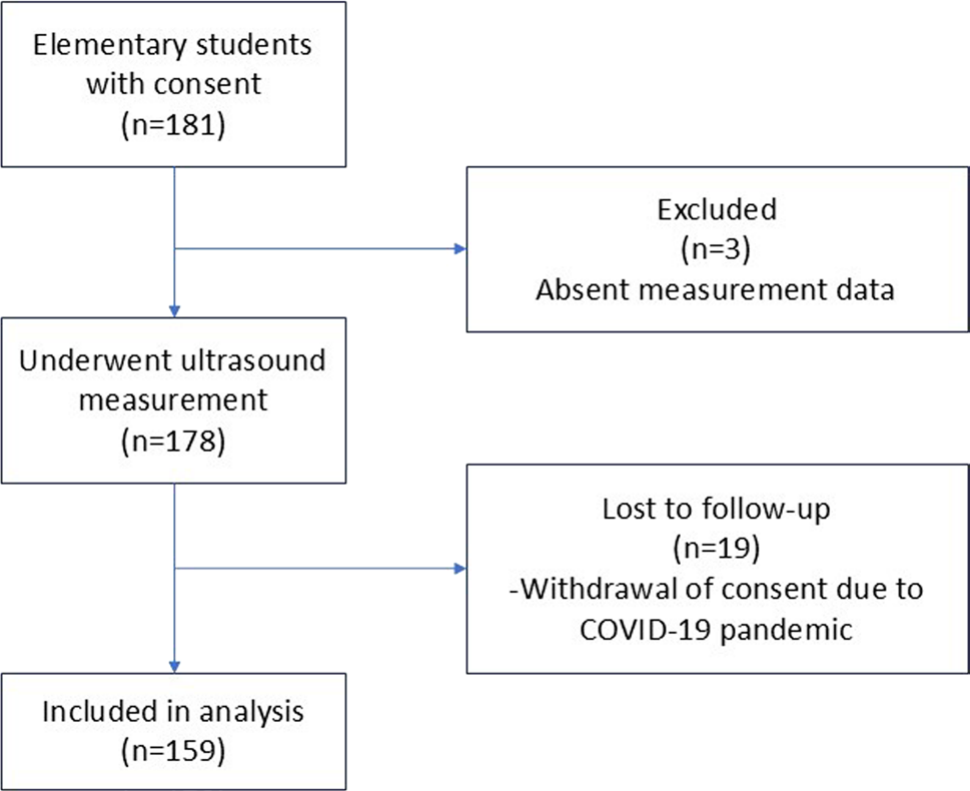
Flow diagram of study participants

Ultrasound measurements of ossification centers were performed on 178 children. Three were absent, and 19 dropped out of height measurements due to the COVID-19 pandemic, resulting in 159 participants for final analysis (Fig. 2).

Table 3 shows the grade level and sex at the time of ultrasound measurement, as well as the follow-up duration and number of height measurements. There were 96 boys and 63 girls, totaling 159 participants. The median follow-up period for height measurements was 5.4 years (25th percentile: 4.4 years, 75th percentile: 6.6 years), with a median of 16.0 measurements (25th percentile: 12.0, 75th percentile: 19.0). The number of height measurements ranged from a minimum of nine (boys: 3 years from first grade to fourth grade, girls: 3.4 years from first grade to fourth grade) to a maximum of 22 (8.6 years from first grade to ninth grade). Of the 159 participants (96 boys, 63 girls), 84 had reached PHVA at the time of analysis (35 boys, 49 girls). Due to the COVID-19 pandemic, measurement frequency decreased in 2021, with elementary school students measured twice a year and junior high school students once a year. Despite variations in follow-up duration and measurement frequency due to absences or transfers, 1–3 measurements were conducted annually.

**Table 3:** Demographic characteristics and follow-up data of participants at the time of ultrasound measurement

	*n* (girls)	Min	Max	Median (IQR)
Grade				
1	34 (11)			
2	28 (9)			
3	27 (13)			
4	29 (17)			
5	21 (7)			
6	20 (6)			
Total	159 (63)			
Follow-up (y)		3.0	8.7	5.4 (4.4–6.6)
Measurements (N)		9.0	22.0	16.0 (12.0–19.0)

n: number of participants, y: number of follow-up years, N: number of measurements

Initially, forced entry multivariable regression analysis was performed. Multicollinearity was observed for the olecranon and calcaneus, which had multiple evaluation sites, with the variance inflation factor (VIF) exceeding 10 (Supplementary Table 1). Therefore, analysis was limited to one site each for the olecranon and calcaneus.

The short axis of the epiphyseal nucleus of the olecranon ossification nucleus may be less reproducible depending on the measurement position and angle, but the long axis cartilage distance is easy to measure, so the long axis cartilage distance was selected.

Previous studies have reported that the ossification nucleus appears by splitting on the Achilles tendon side of the calcaneus [15, 16], and the medial process of the calcaneal tuberosity is sometimes difficult to visualize on the plantar side, so the thickness of the cartilage was selected.

Stepwise multivariable regression analysis was performed on eight bones and eight sites. The results are shown in Table 4.

**Table 4:** Multiple regression analysis results for predicting years to PHVA

	Unstandardized Coefficient		Standardized β	*p*-value	VIF
	β (95% CI)	Std. Error			
(Constant)	1.206 (0.389, 2.022)	0.413		0.004	
Calcaneus plantar thickness	0.562 (0.365, 0.758)	0.099	0.392	0.000	3.454
Plantar sesamoid	− 1.120 (− 1.586, − 0.655)	0.236	− 0.266	0.000	2.239
Tibial tuberosity	− 0.675 (− 1.224, − 0.125)	0.278	− 0.147	0.016	2.618
Hook of the hamate	0.229 (0.042, 0.417)	0.095	0.186	0.017	4.238

*VIF* variance inflation factor

The final prediction equation included four ossification centers: calcaneus plantar (thickness), plantar sesamoid, tibial tuberosity, and hook of the hamate.

The prediction equation: Years to PHV = 1.206 + (0.562 × calcaneus plantar thickness) – (1.120 × plantar sesamoid) – (0.675 × tibial tuberosity) + (0.229 × hook of the hamate).

This model achieved an adjusted *R*^2^ of 0.782.

To facilitate clinical interpretation, example cases demonstrating how ultrasound findings can be used to estimate years to PHV using this equation are presented (Supplementary Fig. 2).

In addition, evaluation of a single ossification center suggested that the thumb sesamoid appears a few months before PHV (Supplementary Figure S3h); Supplementary Figure S3 provides information on other ossification centers.

## Discussion

This study demonstrated that ultrasound evaluation of secondary ossification centers may serve as a useful indicator for predicting years to PHV. To the best of our knowledge, this is the first attempt to use ultrasound to evaluate multiple ossification centers for predicting the timing of PHV. The use of multiple ossification centers resulted in an *R*^2^ of 0.782, suggesting higher prediction accuracy than single ossification center evaluations [8].

The growth of the hook of the hamate can be uniquely observed with ultrasound, providing an advantage over other methods. Its simultaneous visualization with the pisiform bone enhances reproducibility. Previous research has reported that completion of the hook of the hamate occurs a few months before PHV [17], which the results of the present study support, since cartilage thickness of 0–1 mm suggested hook completion (Supplementary Figure S3a).

The calcaneus plantar thickness was easily visualized via longitudinal imaging of the plantar fascia attachment. Previous X-ray studies have shown that the calcaneal ossification center appears about 4.7 years before PHV [15]. The present prediction equation, when inputting “calcaneus plantar (thickness) = 4”, generally showed no plantar sesamoid and tibial tuberosity ossification centers, resulting in years to PHV = 4.1–4.6 years, consistent with previous findings.

For the plantar sesamoid, it was considered “present” if at least one ossification center appeared, since some individuals showed development only on the medial or lateral side, possibly due to differences in weight-bearing surfaces. Previous studies have shown its appearance about 1.35 years before PHV [18], which is consistent with the present univariate analysis results (Supplementary Figure S3k).

Though staging classifications exist for the tibial tuberosity with both X-ray and ultrasound [19, 20], a binary function was chosen to avoid subjective judgments in qualitative assessments. Generally, the secondary ossification center is said to appear around age 9 years for girls and 11 years for boys [21], but no previous studies using it to predict the timing of PHV or skeletal age were found.

Although not included in the final prediction equation, the thumb sesamoid was predicted to appear just before PHV, consistent with previous studies [18, 22, 23], making it a good indicator for PHV timing. However, it is not suitable for prediction because it can only be evaluated at or after PHV. This aspect requires further investigation in future studies.

Though racial differences exist, boys generally reach PHV at 13 years and girls at 11 years, with the interval from take-off age (TOA) to PHVA reported as 2.75 years for boys and 1.87 years for girls [24, 25]. The present equation enables PHVA estimation up to approximately 5 years in advance, allowing growth timing to be predicted even before TOA.

The four selected ossification centers can be rapidly and easily visualized on ultrasound, suggesting potential for easy clinical application without radiation exposure.

Even when assessing all 12 sites across eight bones, the procedure is technically simple, and each site can be scanned within approximately 10 s, requiring only 1–2 min in total. Since this study identified four key ossification centers, the measurement time could be further reduced to approximately 30 s to 1 min. However, additional time is needed for distance measurements on the ultrasound images.

Skeletal maturity assessment methods, including the Greulich–Pyle bone age, Tanner staging, and Sanders classification, have been used to approximate bone age or pubertal development stages. However, these methods were not originally designed to estimate the timing of PHV, and few studies have quantitatively evaluated their performance for this specific purpose [6]. Therefore, future studies should aim to directly compare ultrasound-based methods with these established radiographic standards to evaluate their relative predictive performance.

This ultrasound-based approach may aid in predicting PHV timing, which could be valuable for adjusting exercise intensity and type, as well as for bio-banding to reduce sports injury risk. Moreover, its application is not limited to sports medicine; it may also assist in evaluating bone maturation in conditions such as adolescent idiopathic scoliosis [26, 27].

Beyond skeletal assessment, this method may also provide insights into growth and development. Pubertal timing has been associated with self-esteem, mental health, and social adjustment, with variations in maturation potentially influencing long-term psychological outcomes [28] [29]. Differences in biological maturity within the same age group have been linked to disparities in social confidence and psychological well-being [30]. In sports settings, these variations may affect competition, motivation, and talent identification, suggesting that predicting the timing of PHV could help inform training adaptations and injury prevention strategies [31] [32]. Though further validation is required, early identification of PHV timing may contribute to more individualized approaches to growth monitoring and developmental support.

The present study had some limitations. Most participants were of Asian (Japanese) descent, necessitating validation in other racial groups. The higher proportion of male participants and the lack of consideration of factors such as illnesses, obesity, sports activities, and biological influences such as nutrition, genetics, and hormones may affect the estimation of PHV timing [33, 34]. However, the ability to obtain useful indicators without these factors, prioritizing prediction using only ultrasound with clinical application in mind, is significant. Future studies should validate these findings with new datasets.

The reduced frequency of height measurements due to the COVID-19 pandemic may have affected PHVA calculations. In addition, due to occasional absence, some participants were measured only once or twice per year, while others had three measurements, making clear stratification by measurement frequency infeasible. However, previous studies have predicted PHV using only one or two annual measurements [12, 35], suggesting that the present approach remains robust despite this variation. Though ultrasound evaluation has inherent reproducibility uncertainties, measurement errors were minimized by strictly defining measurement methods and treating variables as continuous or binary functions. However, all ultrasound measurements were performed by a single examiner, which ensured consistency but did not allow for the assessment of intra-observer or inter-observer variability. Future studies should incorporate multiple examiners and perform intraclass correlation coefficient (ICC) analysis to enhance the reliability of ultrasonographic measurements. Furthermore, although the regression model demonstrated good explanatory power (adjusted *R*^2^ = 0.782), internal validation such as cross-validation or split-sample validation was not performed in this study. This limitation should be addressed in future research to mitigate the risk of overfitting and improve the generalizability of the predictive model.

The ultrasound measurement methods and continuous function cutoffs were determined by the researchers due to a lack of previous studies. In addition, not all participants had reached PHV at the time of this analysis. Of the 159 participants, 84 had attained PHV; however, the proportion differed by sex, with a higher percentage of girls reaching PHV than boys. Given that boys tend to reach PHV at a later age, their attainment rate was lower at the time of analysis. Follow-up is still ongoing for participants who can continue, and longer monitoring is necessary to ensure accurate growth trajectory analysis. Future studies will continue to track this cohort to assess their final growth patterns and refine the prediction model accordingly.

Due to sample size constraints, this statistical analysis was performed with mixed sex data. Future studies should increase sample sizes to allow for separate analyses by sex. Furthermore, the present study did not compare the ultrasonographic predictive findings with other established methods for estimating PHV timing, such as radiographic bone age assessment, Tanner staging for sexual maturity rating, or traditional skeletal maturity indicators. This absence of comparative data limits the study’s ability to determine whether the proposed method is more accurate than existing techniques. Future research should include direct comparisons with these established methods to further validate the predictive accuracy of ultrasonographic assessment.

## Conclusion

Stepwise regression identified four bones (calcaneus plantar (thickness), plantar sesamoid, tibial tuberosity, and hook of the hamate) for predicting the timing of PHV, leading to the development of a predictive equation. Though further validation is required before clinical application, the selection of these four easily visualized and clinically applicable sites may facilitate the practical use of ultrasound-based growth prediction.

## Supplementary Information

Below is the link to the electronic supplementary material.

Supplementary file1 (TIF 178 KB)

Supplementary file2 (TIF 251 KB)

Supplementary file3 (TIF 356 KB)

Supplementary file4 (TIF 561 KB)
