# Supplementary figures and images for: Ultrasound-based prediction model for years to peak height velocity using multiple secondary ossification centers
Source: J Med Ultrason (2001). 2025 Oct 24;53(2):199–207. doi: 10.1007/s10396-025-01571-y (PMC13092546; doi:10.1007/s10396-025-01571-y)

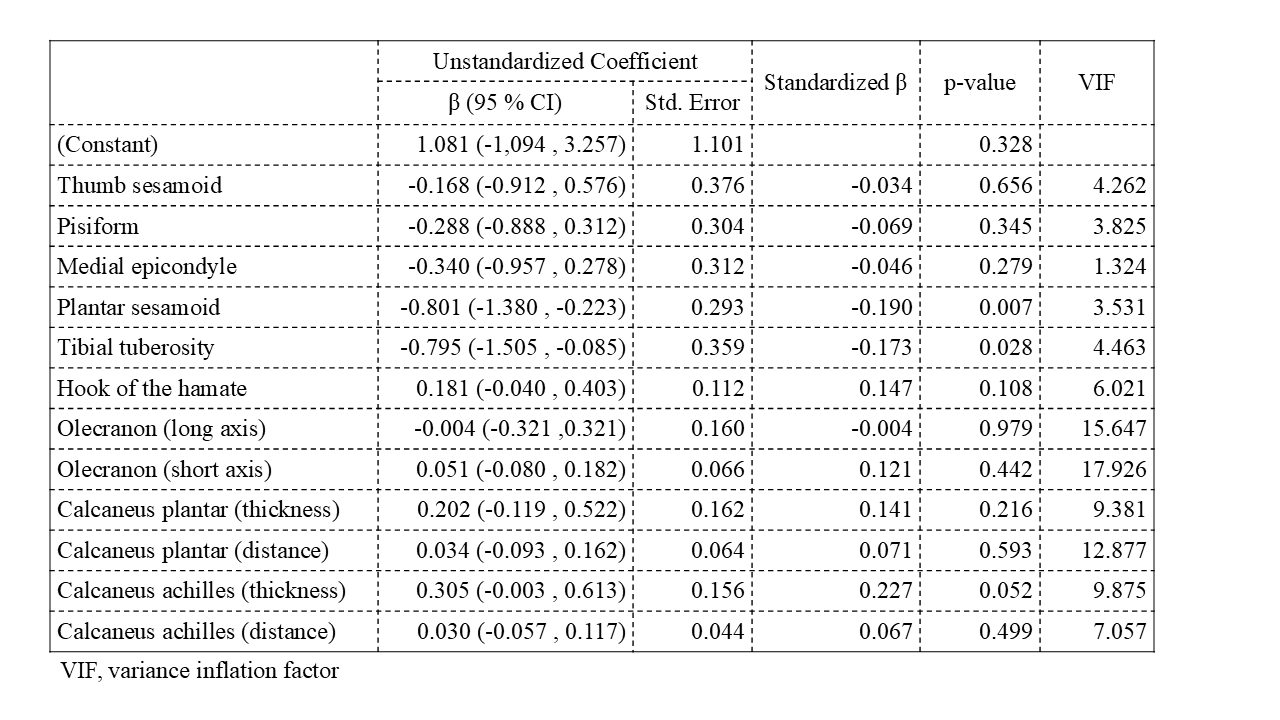

Supplement: Supplementary file 1 — Supplementary file1 (TIF 178 KB) [file 10396_2025_1571_MOESM1_ESM.tif]

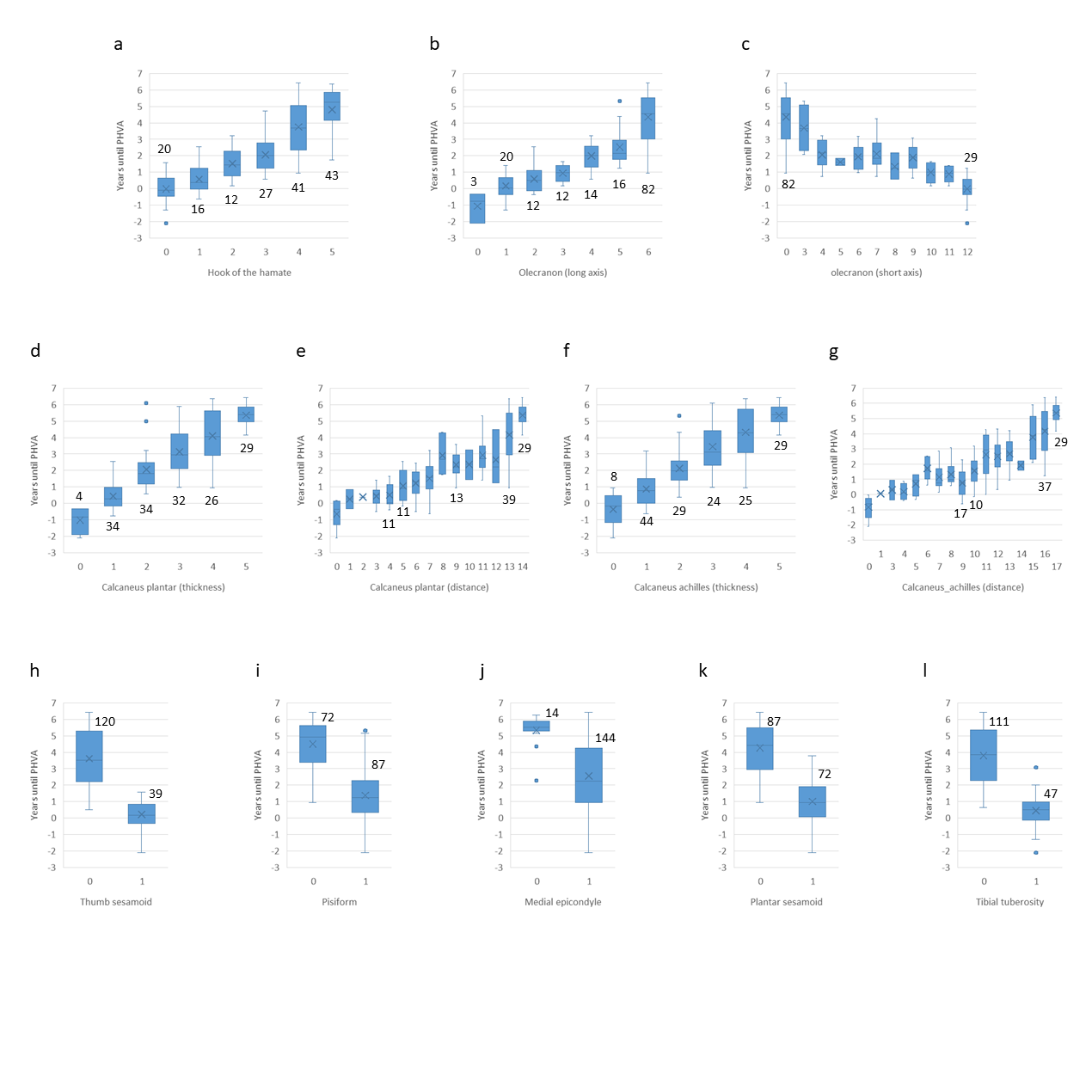

Supplement: Supplementary file 2 — Supplementary file2 (TIF 251 KB) [file 10396_2025_1571_MOESM2_ESM.tif]

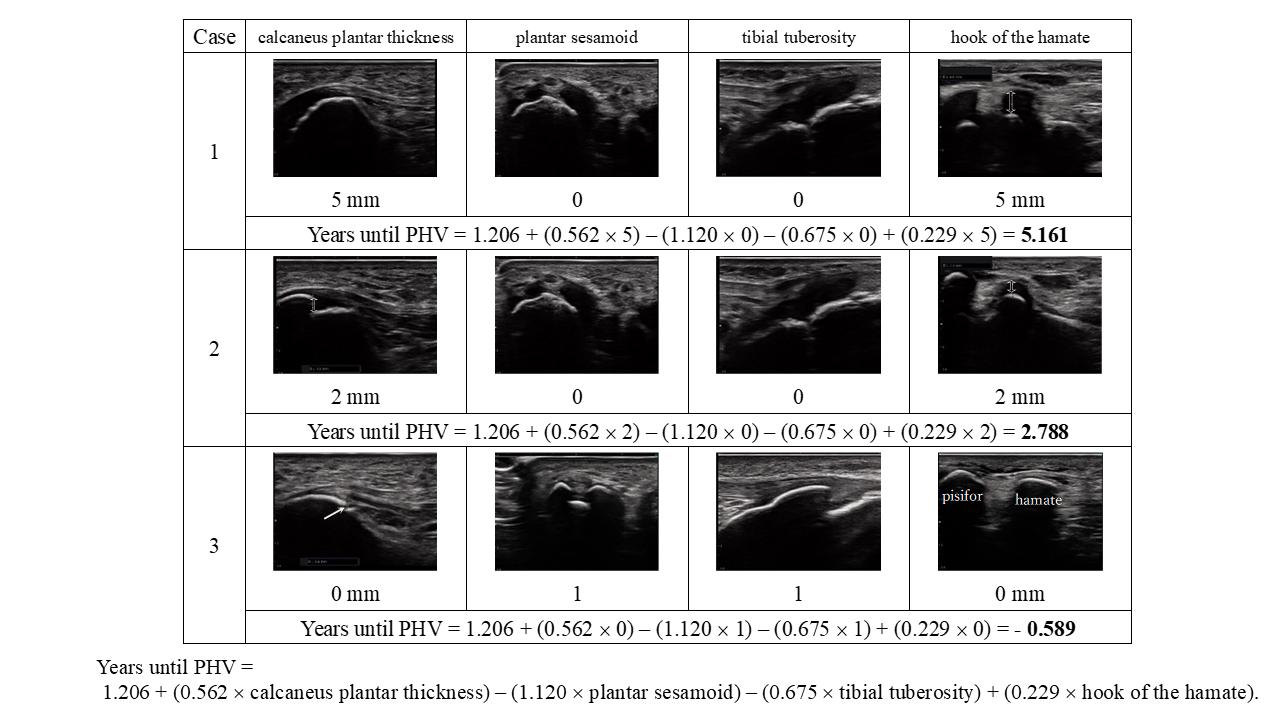

Supplement: Supplementary file 3 — Supplementary file3 (TIF 356 KB) [file 10396_2025_1571_MOESM3_ESM.tif]

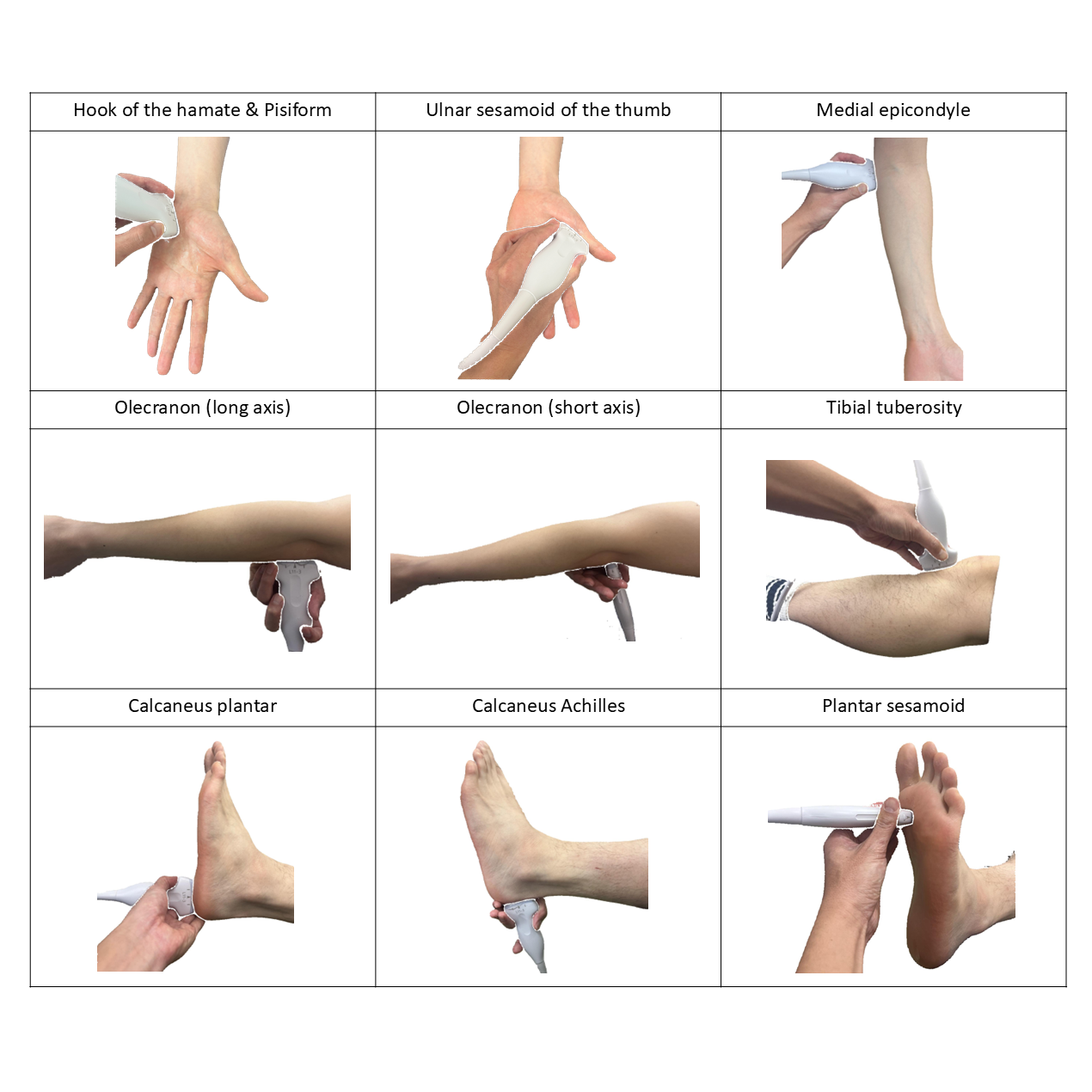

Supplement: Supplementary file 4 — Supplementary file4 (TIF 561 KB) [file 10396_2025_1571_MOESM4_ESM.tif]
